# Effect of Fungal Colonization of Wheat Grains with *Fusarium* spp. on Food Choice, Weight Gain and Mortality of Meal Beetle Larvae (*Tenebrio molitor*)

**DOI:** 10.1371/journal.pone.0100112

**Published:** 2014-06-16

**Authors:** Zhiqing Guo, Katharina Döll, Raana Dastjerdi, Petr Karlovsky, Heinz-Wilhelm Dehne, Boran Altincicek

**Affiliations:** 1 Institute of Crop Science and Resource Conservation (INRES-Phytomedicine), Rheinische Friedrich-Wilhelms-University of Bonn, Bonn, Germany; 2 Molecular Phytopathology and Mycotoxin Research, Georg-August-University Göttingen, Göttingen, Germany; AgroParisTech, France

## Abstract

Species of *Fusarium* have significant agro-economical and human health-related impact by infecting diverse crop plants and synthesizing diverse mycotoxins. Here, we investigated interactions of grain-feeding *Tenebrio molitor* larvae with four grain-colonizing *Fusarium* species on wheat kernels. Since numerous metabolites produced by *Fusarium* spp. are toxic to insects, we tested the hypothesis that the insect senses and avoids *Fusarium*-colonized grains. We found that only kernels colonized with *F. avenaceum* or *Beauveria bassiana* (an insect-pathogenic fungal control) were avoided by the larvae as expected. Kernels colonized with *F. proliferatum, F. poae* or *F. culmorum* attracted *T. molitor* larvae significantly more than control kernels. The avoidance/preference correlated with larval feeding behaviors and weight gain. Interestingly, larvae that had consumed *F. proliferatum-* or *F. poae-*colonized kernels had similar survival rates as control. Larvae fed on *F. culmorum*-, *F. avenaceum*- or *B. bassiana*-colonized kernels had elevated mortality rates. HPLC analyses confirmed the following mycotoxins produced by the fungal strains on the kernels: fumonisins, enniatins and beauvericin by *F. proliferatum*, enniatins and beauvericin by *F. poae,* enniatins by *F. avenaceum,* and deoxynivalenol and zearalenone by *F. culmorum*. Our results indicate that *T. molitor* larvae have the ability to sense potential survival threats of kernels colonized with *F. avenaceum* or *B. bassiana*, but not with *F. culmorum*. Volatiles potentially along with gustatory cues produced by these fungi may represent survival threat signals for the larvae resulting in their avoidance. Although *F. proliferatum* or *F. poae* produced fumonisins, enniatins and beauvericin during kernel colonization, the larvae were able to use those kernels as diet without exhibiting increased mortality. Consumption of *F. avenaceum*-colonized kernels, however, increased larval mortality; these kernels had higher enniatin levels than *F. proliferatum* or *F. poae*-colonized ones suggesting that *T. molitor* can tolerate or metabolize those toxins.

## Introduction

*Fusarium* species (Ascomycota, Nectriaceae) are among the most diverse and widespread plant-infecting fungi [1]. They cause important diseases of maize, small-grain cereals, vegetables and even trees [2]. Decreased yield as well as diminished quality of plant products due to *Fusarium* infection cause significant economic losses worldwide [3], [4]. Moreover, *Fusarium* species are prominent producers of medically relevant mycotoxins [5], [6]. Toxicologically most important mycotoxins produced by *Fusarium* species comprise sesquiterpenoids trichothecenes such as T-2 toxin and deoxynivalenol [7], polyketides such as fumonisins [8], and depsipeptides such as beauvericin or enniatins [9], [10]. Mycotoxin exposure resulting from the ingestion of contaminated products poses a hazard to human and animal health [3], [11], [12]. Moreover, immunocompromized patients occasionally develop invasive fusariosis caused most often by *F. solani*, *F. oxysporum,* or *F. verticillioides* [13], [14] and some mycotoxins were found to suppress humoral as well as cell-mediated immunity in mammals [11], [12].

Beauvericin a cyclic hexadepsipeptide was isolated from an entomopathogenic fungus *Beauveria bassiana* and was demonstrated to be toxic to invertebrates [15] before it was identified in extracts of two *Fusarium* species that were toxic to Colorado potato beetle [16]. Toxicity of beauvericin to insects is now well established [10], [17]. Enniatins, chemically closely related to beauvericins, were purified from extracts of *Fusarium* species because of their antibiotic activity [18]. Insecticidal properties of enniatins were discovered only after enniatins were purified from cultures of entomopathogenic (and plant pathogenic) species *Fusarium lateritium* [17]. The demonstration of the toxicity of enniatins was later extended to further insect species [19]. Although beauvericin and enniatins are most prominent insecticidal mycotoxins of *Fusarium* species, toxic effects of other *Fusarium* mycotoxins on insect individuals as well as tissue cultures were reported [20]–[22]. Studies performed with purified mycotoxins are inherently limited because toxic effects in nature result from exposures to mixtures of compounds with additive or synergistic effects, involving known mycotoxins as well as numerous less- or unknown metabolites. Moreover, while the biological function of trichothecenes as virulence factors in plant infection [23] and zearalenone as agent of interference competition and protection against mycoparasitic fungi [24] have been demonstrated, little is known about the biological functions and ecological roles of numerous other *Fusarium*-mycotoxins.

With few exceptions, *Fusarium* species are not known as entomopathogens [22] and our understanding of ecological interactions of grain-feeding insects and grain-colonizing fungi is still scarce. Here, we used *T. molitor* to investigate interactions of meal beetles with diverse *Fusarium* species on wheat kernels. *B. bassiana* is a potent pathogen of tenebroid beetles [25] and was used as a positive control and uninfected kernels were used as negative control. *T. molitor* is an important and globally distributed pest of stored products and its capability of selecting optimal ratios of dietary components [26] indicates that beetles may also sense and avoid toxic fungi-colonized diet. Our working hypothesis was that *T. molitor* can distinguish among kernels colonized with diverse *Fusarium* species or *B. bassiana* and that the insect’s repulsion/attraction of respective kernels correlates with their impact on larvae’s survival.

## Materials and Methods

### Ethics Statement

No specific permits were required for the studies described here: a) no specific permissions were required for these locations/activities; b) locations were not privately-owned or protected; c) the studies did not involve endangered or protected species.

### Study System

Isolation and identification of the strains was described in a previous study [27]. In brief, *F. avenaceum* 1.27 was isolated from colonized wheat kernels in the year 2008 at Poppelsdorf, Bonn, Germany, and taxonomically characterized as described in this study; *F. culmorum* 3.37 was isolated from colonized wheat in the year 2004 at Klein-Altendorf, Bonn, Germany; *F. poae* DSM 62376 was purchased from Deutsche Sammlung von Mikroorganismen und Zellkulturen (DSMZ, Braunschweig, Germany); *F. proliferatum* 21.1 was isolated from colonized maize in the year 2007 at Hainichen, Germany; and *B. bassiana Bea2* was isolated from infected *Otiorhynchus sulcatus* (the black vine weevil) in the year 1989 at Stuttgart, Germany. The strains were grown on potato dextrose agar (PDA) plates in darkness at 23°C.

The meal beetle *T. molitor* is a common storage pest and destructive insect species. *T. molitor* larvae were reared on whole wheat flour with 5% yeast extract in a climate chamber in darkness at 27±2°C and a relative humidity of 65±5%. Last instar larvae were starved for 72 h and were randomly selected prior use in experiments.

### Inoculation of Wheat Kernels

For preparing diet contaminated with fungi, spring wheat kernels (cultivar Taifun) were soaked in distilled water for 18 h at room temperature and placed into 1 litre plastic bags separately, subsequently autoclaved for 30 min. Autoclaved wheat kernels were inoculated with fungal mycelia on PDA agar and incubated at room temperature for 4 weeks to ensure complete colonization of the kernels. Control diet was handled in a same way with un-inoculated PDA agar.

### Larvae’s Preference/Avoidance Experiments

To determine preference or avoidance reactions of the larvae, feeding experiments were performed on Petri dishes with a diameter of 142 mm. The dishes were marked to generate four equal sectors in the form of identical pie slices (1, 2, 3 and 4). Into opposing sectors each 6 g uninfected kernels were placed and in the remaining two sectors each 6 g of kernels of interest (fungi-colonized or non-colonized). Then, 10 individuals of *T. molitor* were placed randomly in the centre of the Petri dishes. After 20 min in darkness and without any disturbance, the number of larvae in each sector was determined. In total, 20 repetitions with using each 10 naive, inexperienced larvae per sample were performed within one replicate and in total three independent replicates were performed.

### Larval Weight Gain Determination

To monitor feeding of larvae, we determined the cumulative weight gain of 10 larvae within 24 h when fed on fungi-colonized or control kernels. Integral weights of 10 individuals were measured before and 24 h after placing on respective kernels. Per treatment 10 independent determinations were performed and in total three independent replicates were performed.

### Survival Rate Analysis of Larvae on Fungal Mycelium, Colonized Kernels or upon Stabbing or Injection-based Infections

Per treatment 30 individuals of *T. molitor* were reared on mycelia of diverse *Fusarium* strains grown on PDA plates and survival rates were monitored daily for 15 days. In total five independent replicates were performed. For survival analysis on fungi-colonized kernels, 30 individuals of *T. molitor* per treatment were reared on respective kernels and survival rates were monitored for 15 days. In total three independent replicates were performed. For stabbing-based infection experiments, *F. avenaceum*, *F. culmorum*, *F. poae*, *F. proliferatum* or *B. bassiana* were inoculated on PDA plates and incubated for 2 weeks. Sterilized insect minutin pins were used to scratch a mixture of mycelia and conidia from the plate and to wound larvae dorso-laterally leaving mycelia and spores as small plug at the wounding site. In three independent replicates survival of larvae was monitored for the period of one week. Additionally, infection with spores using a syringe-based injection of approx. 10^4^ conidia into each larva in 5 µl of water with 0.01% Tween 20 was performed. Survival of larvae was monitored after 5 days incubation in three independent replicates, with 120 larvae per group and per replicate. Living larvae were harvested at 0 day, 5 days, 10 days and 15 days time points then freeze-dried at −50°C for 48 h. The freeze-dried larvae were ground for subsequent DNA extraction and quantitative real time PCR analysis.

### Mycotoxin Analysis

Wheat kernels and larvae were freeze-dried, ground and extracted as described [28]. Samples analyzed for beauvericin and enniatin content were not defatted to avoid losses of the mycotoxins in the organic phase. HPLC separation was performed on a RP column at 40°C and trichothecenes A, B and zearalenone were detected by tandem mass spectrometry using triple quadrupol 1200 L (Varian, Darmstadt, Germany) based on published methods [29], [30]. Two mass transitions were used for each toxin. Beauvericin, fumonisin B1 and enniatins were separated on the same HPLC system but detected using ion trap 500 MS (Varian, Darmstadt, Germany) as described [28]. For each mycotoxin detected on the ion trap, three mass transitions were used. Calibration curves were constructed using analytical standards dissolved in methanol/water (1∶1) with a correction for recovery and matrix effects. The limits of quantification for deoxynivalenol, nivalenol, fusarenon X, T2-toxin, diacetoxyscirpenol and neosolaniol, beauvericin, enniatin A, B, A1, B1 and fumonisin B1 ranged between 9 and 170 µg/kg in kernels and 9 and 130 µg/kg in larvae.

### DNA Extraction, Sequencing and Real-time PCR

qPCR was conducted to monitor if any of tested *Fusarium* strains had the ability to proliferate in *Tenebrio* body cavity or tissue. Total DNA from *T. molitor* larvae was extracted from 30–50 mg of freeze-dried material using a CTAB method [31], purified by phenol extraction, precipitated with isopropanol and dissolved in 50 µl TE buffer (10 mM Tris, 1 mM EDTA, pH 8.0). DNA was diluted fifty fold prior to PCR analysis.

Thermocycler (CFX384™, BioRad, USA) was used for real-time PCR analysis (qPCR) in a total volume of 4 µl. Primers MGBF/R [32], OPT 18F/R [33], Fp 82F/R [34], Fp3F/4R [35] and P1/P3 [36] were used for *F. avenaceum*, *F. culmorum*, *F. poae, F. proliferatum* and *B. bassiana*, respectively. The reaction mixture consisted of buffer (16 mM (NH4)_2_SO_4_; 67 mM Tris-HCl; 0.01% (v/v) Tween-20, pH 8.8 at 25°C, Bioline, Lükenwalde, Germany), 0.15 mM of each dNTP (Bioline, Lükenwalde, Germany), 2.5 mM MgCl_2_, 0.1 U of *Taq* DNA polymerase (BIOTaq, Bioline, Lükenwalde, Germany), 0.3 µM of each primer, 0.1×SYBR Green I (Invitrogen, Karlsruhe, Germany) and 1 mg/ml bovine serum albumin. The lowest standards set as limits of quantification (LOQ) were 14–40 ng/g for all four *Fusarium* spp. and 1.0 µg/g for *B. bassiana*.

To amplify translation elongation factor 1-alpha (TEF) gene region, primers ef1/ef2 [37] were used. PCR was performed in reaction mixture described above with hot-start DNA polymerase (Immolase DNA Pol, Lükenwalde, Germany) in a total volume of 25 µl The following cycling conditions were used: 1 cycle of 10 min at 95°C, 30 cycles of 60 s at 94°C, 45 s at 58.5°C, and 60 s at 72°C, followed by a final extension cycle at 72°C for 5 min. Amplified DNA products were sequenced (LGC Genomics, Berlin, Germany) by Sanger method in both directions. The sequence of the TEF1a gene of *F. avenaceum* strain 1.27 was deposited at EMBL Nucleotide Sequence Database with the accession number HG794242. The taxonomical identity of the strain was determined with the help of morphological characters [38], its mycotoxin profile [6] and TEF1a sequence [39].

### Statistical Analyses

Statistical analyses were performed with R 2.15.3 [40]. Weight gain values were log transformed before ANOVA. Survival rates over time were analyzed with a Cox regression model (coxph function) and survival proportion values were fitted to a generalized linear model (GLM) with quasibinominal error structure and logit link function.

## Results

### Survival Rates of *Tenebrio* Larvae Fed on Mycelium of *Fusarium* Species

**Figure 1:**
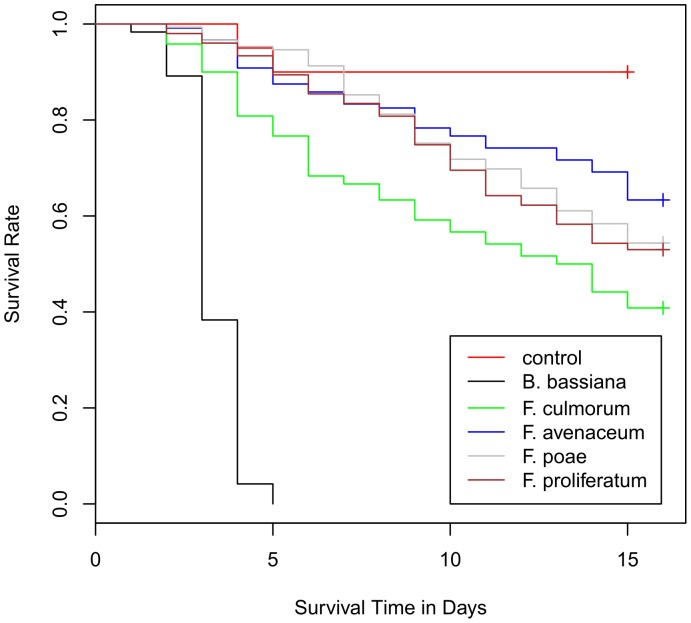
Survival of larvae feeding on mycelium of diverse *Fusarium* species grown on PDA. Survival curves of larvae reared on PDA plates covered with mycelium of diverse *Fusarium* species were significantly reduced when compared to controls (Cox regression model, χ^2^ = 35.53; d.f. = 8; P<0.001; N = 560). In addition, survival curve on mycelium of the entomopathogen *B. bassiana* is shown as positive control.

In a first step we determined the capability of *Fusarium* species to induce mortality of larvae. We monitored the survival rates of *T. molitor* larvae reared on PDA plates covered with mycelium of four *Fusarium* species during a period of 15 days. Under these conditions fungal mycelium on PDA was the sole diet available for the larvae. We fitted a Cox’s proportional hazards model with censoring on the data set and found evidence for significant differences between the survival curves (log-rank-test, χ^2^ = 35.53; d.f. = 8; P<0.001) with a significant effect of *Fusarium* strains (χ^2^ = 27.17; d.f. = 4; P<0.001) when compared to controls (Fig. 1). We also found a significant effect of the second variable replicate (χ^2^ = 10.3; d.f. = 4; P = 0.036) with one out of five replicates showing some difference (z = 2.01; P = 0.045) when compared to the other replicates. Our analysis revealed that the daily hazard of larvae survival increased by 7.85 times (confidence interval at 95% level (CI) = 1.89 to 32.65 times) when feeding on *F. culmorum* mycelium, by 3.76 times (95% CI = 0.89 to 15.81 times) on *F. avenaceum* mycelium, by 4.96 times (95% CI = 1.19 to 20.76 times) on *F. poae* mycelium and by 5.34 times (95% CI = 1.28 to 22.29 times) on *F. proliferatum* mycelium, respectively, when compared to controls.

### Larval Preference or Avoidance to Fungi-colonized Wheat Kernels

**Figure 2:**
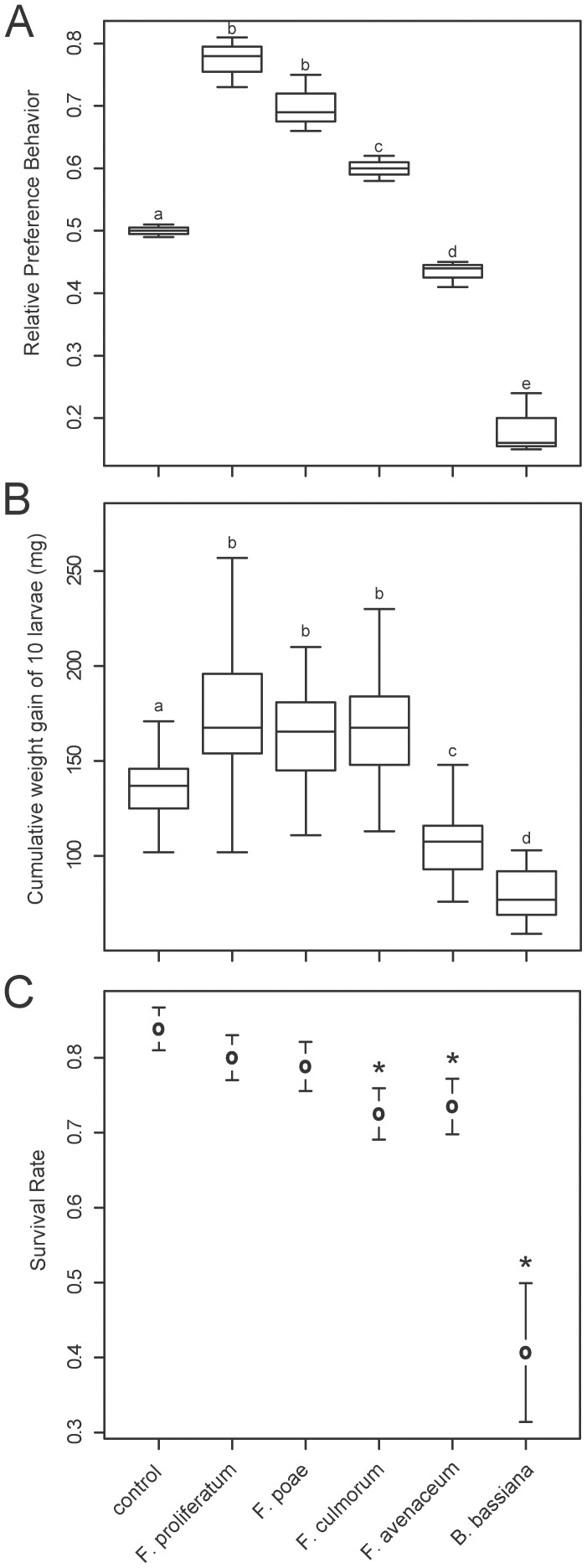
Reactions of larvae to fungi-colonized wheat kernels. (A) Boxplot of relative preference or avoidance reactions of larvae within 20 min towards fungi-colonized wheat kernels. N = 360. (B) Boxplot of cumulative weight gain of each 10 larvae per data point within 24 h on colonized wheat kernels with in total N = 1,800. (C) Relative survival rate of larvae on colonized kernels was determined within 15 days. Results are shown as mean values ± CI at 95% levels with N = 360. Significant differences are indicated by letters or by asterisks.

In a next step we investigated potential selective feeding behaviors of the larvae as we expected avoidance reactions towards kernels that were colonized with different *Fusarium* species. *T. molitor* larvae showed significant preference or avoidance reactions to colonized kernels depending on the fungal strain (Fig. 2A). Inspection of diagnostic plots as well as use of the Fligner-Killeen test for equality of variances [41] revealed that values were normally distributed and that there was homogeneity of variance for the examined groups. Therefore, an ANOVA test was performed on the data set. Unexpectedly, we found that larvae significantly preferred wheat kernels colonized with *F. proliferatum* (mean ± S.D. = 77±4%; CI = +21 to +33%), *F. poae* (70±5%; CI = +14 to +26%) or *F. culmorum* (60±2%; CI = +4 to +16%) while avoided kernels colonized by *F. avenaceum* (43±2%; CI = −7 to −13%) or *B. bassiana* (18±5%; CI = −26 to −38%) when compared to control kernels (50±1%; CI = 46 to 54%) (ANOVA, F = 113.3; d.f. = 5 and 12; P<0.001).

The preference or avoidance behaviors correlated with significant changes in larval weight gain within 24 h on kernels (Fig. 2B). Inspection of diagnostic plots as well as use of the Fligner-Killeen test for equality of variances revealed that logarithmically transformed values were normally distributed and that there was homogeneity of variance for the examined groups. Therefore, an ANOVA test was performed on the data set with the log-transformed response variable weight gain. When compared to larvae reared on control kernels (cumulative weight gain of 10 larvae within 24 h: 138.1±22.1 mg; CI = 132.2 to 153.4 mg) larvae significantly gained more weight on wheat kernels colonized by *F. proliferatum* (173±35.2 mg), *F. poae* (168.4±32 mg) or *F. culmorum* (167.8±29.1 mg), while gained less weight on kernels colonized by *F. avenaceum* (108.5±20.6 mg) or *B. bassiana* (79.1±13.6 mg) (ANOVA, F = 66.79, d.f. = 7 and 172, P<0.001). Of note, we found a significant effect of the explanatory variable replicate (F = 3.31; d.f. = 2; P = 0.039) with one out of three replicates showing significant difference (t value = −2.57; P = 0.011) when compared to the other two replicates.

### Survival Rates on Wheat Kernels Colonized with Mycotoxin-producing Fungi

We determined the survival rate of larvae ingesting fungi-colonized kernels for a period of 15 days. Fitting the data to a generalized linear model with quasibinominal error structure and logit link function revealed that the survival rate of the *T. molitor* larvae feeding on *F. proliferatum* (80±12%; odds ratio (OR) = 0.77 and CI = 0.53 to 1.13) or *F. poae* (79±13%; OR = 0.72 and CI = 0.49 to 1.05) colonized kernels were not significantly different when compared to survival rates on control kernels (84±11%), even when there was a slight reduction (Fig. 2C). However, survival rates of larvae feeding on *F. avenaceum* (73±14%; OR = 0.53 and CI = 0.37 to 0.77; P<0.001), *F. culmorum* (73±13%; OR = 0.51 and CI = 0.35 to 0.73; P<0.001) or *B. bassiana* (41±36%; OR = 0.13 and CI = 0.09 to 0.19; P<0.001) colonized kernels were significantly reduced when compared to larvae feeding on control kernels (Fig. 2C) (GLM, F = 40.635; d.f. = 5 and 354; P<0.001).

To test our initial hypothesis that larvae’s avoidance levels against fungi-colonized kernels correlate with the capability of respective kernels to induce higher mortality rates in larvae, we performed a Kendall’s tau statistical analysis. This analysis estimates a rank-based measure of association of not normally distributed values. The results of this test indicated that avoidance levels of larvae were correlated with their survival rates in our examined cases (Kendall’s tau factor = −0.71; z = −1.8321, P = 0.033) (Figure S1).

### Mycotoxin Analysis

To examine whether mycotoxins accumulated in colonized kernels were responsible for the reduction of survival rates and avoidance behavior towards colonized kernels, we estimated the content of mycotoxins in wheat kernels and in larvae that died within 15 days. The analysis of kernels confirmed the production of fumonisin B1, enniatins and beauvericin by *F. proliferatum*, of enniatins and beauvericin by *F. poae,* enniatins by *F. avenaceum,* beauvericin by *B. bassiana*, and deoxynivalenol and zearalenone by *F. culmorum* (Table 1). Of note, low amounts of enniatins were found in kernels colonized with *B. bassiana* that likely originated from naturally contaminated kernels. In general, mycotoxins found in kernels were also found in larvae fed on the respective kernels, with the only exception of deoxynivalenol.

**Table 1:** Mycotoxin content in kernels or *T. molitor* larvae.

	*B. bassiana*		*F. avenaceum*		*F. culmorum*		*F. poae*		*F. proliferatum*	
	Kernels	Larvae	Kernels	Larvae	Kernels	Larvae	Kernels	Larvae	Kernels	Larvae
	(µg mycotoxin/g meal)									
Beauvericin	0.03	<LOQ	<LOQ	<LOQ	–	–	30.10	1.19±0.32	36.36	0.24±0.34
Enniatin A	<LOQ	<LOQ	14.26	0.04±0.03	–	–	<LOQ	<LOQ	0.03	0.01±0.01
Enniatin A1	<LOQ	<LOQ	60.51	0.33±0.25	–	–	<LOQ	<LOQ	<LOQ	<LOQ
Enniatin B	0.21	0.19±0.04	>90	13.00±5.96	–	–	0.32	1.84±2.59	27.09	0.09±0.06
Enniatin B1	<LOQ	<LOQ	>90	0.99±0.35	–	–	0.03	0.21±0.31	2.89	0.01±0.01
Fumonisin B1	<LOQ	<LOQ	<LOQ	<LOQ	–	–	<LOQ	<LOQ	39.74	1.86±0.64
Diacetoxyscirpenol	–	–	<LOQ	<LOQ	–	–	<LOQ	<LOQ	–	–
Neosolaniol	–	–	<LOQ	<LOQ	–	–	<LOQ	<LOQ	–	–
T-2 toxin	–	–	<LOQ	<LOQ	–	–	<LOQ	<LOQ	–	–
Deoxynivalenol	–	–	<LOQ	<LOQ	10.24	<LOQ	<LOQ	<LOQ	–	–
Nivalenol	–	–	<LOQ	<LOQ	<LOQ	<LOQ	<LOQ	<LOQ	–	–
Zearalenone	–	–	<LOQ	<LOQ	>210	0.02±0.01	<LOQ	<LOQ	–	–
Fusarenon X	–	–	<LOQ	<LOQ	<LOQ	<LOQ	<LOQ	<LOQ	–	–

The values indicate mean values and respective standard deviation. Hyphens indicate that the mycotoxin was not analyzed.

### Altered Feeding Behavior of Larvae on Colonized Kernels

**Figure 3:**
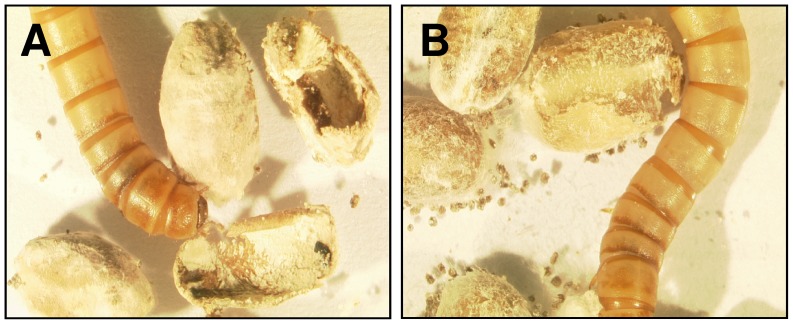
Selective feeding behavior of larvae on fungi-colonized kernels. (A) *F. culmorum* and (B) *F. proliferatum* colonized kernels are shown. Larvae preferred feeding on the inner parts of the kernels infested with *F. culmorum* and on the outer parts of the kernels infested with *F. proliferatum*.

Interestingly, we observed that *T. molitor* larvae showed varying preference in feeding outer or inner parts of colonized kernels, probably as consequence of concentration variations of more water soluble or insoluble fungal metabolites within the kernels (Fig. 3). To quantify these preferences of larvae, we first compared values of respective percentages of kernels with visible feeding traces for all groups. We fitted a generalized linear model with quasibinominal error structure and logit link function to the data and observed that there were significantly more wheat kernels with feeding sign when colonized with *F. proliferatum* (62.93±3.28%; OR = 1.8; CI = 1.56 to 2.06), *F. poae* (61.55±2.69%; OR = 1.69; CI = 1.47 to 1.94) or *F. culmorum* (59.18±6.49%; OR = 1.53; CI = 1.33 to 1.76) and less kernels with sign of feeding colonized with *F. avenaceum* (31.67±3.32%; OR = 0.49; CI = 0.43 to 0.56) or *B. bassiana* (20.29±2.57%; OR = 0.27; CI = 0.23 to 0.31) when compared to controls (48.6±2.22%) (GLM, F = 216.32; d.f. = 5 and 54; P<0.001). Next, we compared kernels with feeding signs divided by the number of kernels with additional signs of caving. Since uninfected kernels as well as kernels colonized with *F. avenaceum* or *B. bassiana* showed no signs of caving at all, these groups were excluded from the analysis. Using a generalized linear model with quasibinominal error structure and logit link function we found significant differences between the groups (GLM, F = 946.31; d.f. = 2 and 27; P<0.001); kernels colonized with *F. proliferatum* with no signs of caving were 92.1±1.99% (OR = 19.05; CI = 15.85 to 23.04), with *F. poae* 90.43±1.83% (OR = 15.46; CI = 13.01 to 18.47) and with *F. culmorum* were 37.93±3.71% (OR = 0.61; CI = 0.56 to 0.67), respectively.

### Survival Rates of Larvae Infected by Inoculation of Conidia into the Hemocoel

To address the question whether *Fusarium* species are capable of replicating in the insect’s hemocoel and thereby contributing to the reduction of larvae’s survival rate we injected fungal strains into larvae. As a first experiment, we performed a stabbing-based infection route using fungi-contaminated needles. Using this approach, we found no significant effect on larval survival rates by injected *Fusarium* strains; however, as expected, a significantly reduced survival rate by entomopathogenic *B. bassiana* (GLM, F = 17.66; d.f. = 6; P<0.001) (Figure S2).

In a second experiment, we used a syringe-based injection method resulting in an inoculation with approx. 10^4^ fungal conidia per larva. Using this second approach with a relatively high infection dose, we determined a significant reduction in larvae’s survival rate post injection with each fungus. Fitting the data to a generalized linear model with quasibinominal error structure and logit link function revealed that larvae’s survival rate was reduced by *F. proliferatum* (65.19±1.7%; OR = 0.53; CI = 0.42 to 0.67), *F. poae* (66.3±2.31%; OR = 0.56; CI = 0.45 to 0.71), *F. culmorum* (29.63±3.57%; OR = 0.12; CI = 0.09 to 0.15) or *F. avenaceum* (32.6±2.31%; OR = 0.14; CI = 0.11 to 0.17) when compared to wounded controls (77.78±5.09%) or sterile puffer injected animals (74.81±1.69%) (GLM, F = 300.32; d.f. = 6 and 14; P<0.001). Values from *B. bassiana* challenged larvae were excluded from the analysis, since all treated animals died resulting in a variance of 0 thereby disturbing statistical analysis.

The DNA content of used *Fusarium* strains in inoculated larvae was estimated by qPCR after a period of 5, 10, and 15 days to determine whether the fungi can germinate and grow in the larvae. We were unable to detect DNA of any of the tested *Fusarium* species in living larvae; only DNA of *B. bassiana* used as a positive control was measurable (Table 2).

**Table 2:** Fungal DNA in larvae injected with conidia.

Fungal species	Species-specific fungal DNA in larvae (µg/g)			
	Days post injection(day)			
	0	5	10	15
*Beauveria bassiana*	<LOQ	57±75	130±87	118±88
*Fusarium avenaceum*	<LOQ	<LOQ	<LOQ	<LOQ
*Fusarium culmorum*	<LOQ	<LOQ	<LOQ	<LOQ
*Fusarium poae*	<LOQ	<LOQ	<LOQ	<LOQ
*Fusarium proliferatum*	<LOQ	<LOQ	<LOQ	<LOQ

Means and standard deviations are shown. The limits of quantification (LOQ) values were 14–40 ng/g for *Fusarium* spp. and 1.0 µg/g for *B. bassiana*.

## Discussion

The present study provides insights into ecological interactions of the meal beetle *T. molitor* with selected *Fusarium* species and *B. bassiana* on wheat kernels. When fungal mycelium was provided as the only food source, survival rates of the larvae were reduced with all tested *Fusarium* species. However, in a more ecologically relevant situation, when fungus-colonized wheat kernels were used as diet, we observed that the survival rates of *T. molitor* larvae fed on *F. proliferatum*- or *F. poae*-colonized kernels were similar to controls and larvae fed on *F. avenaceum*-, *F. culmorum*- or *B. bassiana*-colonized kernels were significantly reduced when compared to survival rates of larvae fed on non-colonized kernels. *T. molitor* larvae preferred feeding on kernels colonized with *F. proliferatum*, *F. poae,* or *F. culmorum* over control kernels and avoided feeding on kernels colonized with *F. avenaceum* or *B. bassiana*. These behavioral reactions correlated with the capability of fungal species to reduce larvae survival on colonized wheat kernels, except for *F. culmorum-*colonized kernels.

None of the tested *Fusarium* species multiplied in living larvae when injected into the insect’s hemocoel, in contrast to the entomopathogen *B. bassiana*. The differences within some independent replicates in our experiments provide some evidence for the hypothesis that *Fusarium* mycotoxins are responsible for increased insect mortality rates, since mycotoxin levels tend to vary between replicates even if environmental factors are strictly controlled [42].

Larve fed on grain colonized with *F. culmorum* exhibited the highest mortality rate. Major mycotoxins produced by *F. culmorum* are deoxynivalenol and zearalenone. High levels of both mycotoxins were found in kernels colonized with *F. culmorum* but only low or undetectable concentrations were found in larvae fed on *F. culmorum*-colonized kernels. This may be a result of insects avoiding kernel parts with high toxin content and/or that these mycotoxins were efficiently metabolized. However, the survival of larvae fed on these kernels was low indicating that either transformation products of deoxynivalenol or zearalenone were still toxic or other toxic products of *F. culmorum* were present. In *F. avenaceum*-colonized kernels high levels of enniatins may have contributed to the elevated mortality rates of larvae fed on colonized kernels; insect toxicity of enniatins is well documented [17], [19]. Interestingly, we detected only low amounts of beauvericin in wheat kernels colonized with *B. bassiana,* which probably killed insects by utilizing other mechanisms including multiplication within the host. Another evidence that beauvericin may not be responsible for larval mortality in this study is the high survival rates of larvae fed on *F. proliferatum* and *F. poae*-colonized kernels, which contained high levels of beauvericin.

Natural selection is likely to act on multiple levels in both insects and fungi. Insects may use fungi as diet or may avoid potentially pathogenic fungi or fungal toxins. For example, the beetle *Coccinella septempunctata* a predator of many insects was shown to avoid *B. bassiana*-colonized insect preys or contaminated leaf surfaces [43]. A similar avoidance behavior was found in the bug *Anthocoris nemorum* [44]. Fungal volatiles represent key repellence signals for these insects and probably also for *T. molitor* in our study. A recent study with the termite *Macrotermes michealseni* provided evidence that a mixture of the volatiles 4, 5-dihydro-5-pentyl-2-(3H)furanone, borneol, 4-nonanone, 2-nonanone, butyrolactone, and camphor contributed largley to the repellency of *B. bassiana* to this termite species [45]. On the other side, insects may increase their tolerance or resistance against potentially pathogenic fungi or their toxins. Supporting this view, larvae of *T. molitor* have recently been reported to be much more tolerant or resistant to tested *Fusarium* mycotoxins than other insect larvae such as armyworms; Moore and Davis reported that *T. molitor* larvae were about 100 times more tolerant to dietary T-2 toxin than the armyworm *Mamestra configurata* [46]. Genomic data of a related tenebrionid beetle, the red flour beetle *Tribolium castaneum,* further supports this view by identifying specific genetic adaptations including gene duplications of the CYP450 subfamilies CYP6 and CYP9, which are known to be involved in toxin resistances [47], or of immune-inducible anti-fungal thaumatins [48]. Taken together, this indicates that tenebrionid beetles may have adapted to counteract to toxic fungi in their environments and determination of the genome sequence of *T. molitor* may further help to address this hypothesis. Fungi, on the other side, may produce chemical compounds to react to competition or may increase tolerance against grazing. Moreover, producing diverse volatile components fungi could easily gain fitness benefits by attracting, repelling or remaining invisible to potential insect hosts, competitors or vectors depending on selective pressures. Indeed, recent studies revealed the role of fungal toxins in interactions of fungi with fungivorous arthropods [49]–[52]. Enhanced production of fungal toxins as a defense response of *Aspergillus nidulans* against fungivorous collembolans was recently established [53]. In the present study we have not investigated induced reactions of *Fusarium* fungi to the presence or feeding of beetles; this will be the subject of subsequent studies.

In conclusion, our study shed light on ecological interactions of *T. molitor* larvae with four *Fusarium* species. We found that larvae have evolved to sense threats derived from *F. avenaceum-* or *B. bassiana*-colonized kernels, but not the threats of *F. culmorum*-colonized kernels. Kernels infested with *F. poae* or *F. proliferatum* showed no significant impact on larvae’s survival rates under tested conditions. To identify the nature of the threat signals for the larvae will be part of our future studies. Knowledge on these signals as well as their correlations with the presence of specific mycotoxins may help better understand mutual ecological adaptations of meal beetle and *Fusarium* species.

## Supporting Information

Figure S1 **Correlation of avoidance levels of larvae towards fungi-colonized kernels to their respective survival rates.**
(TIF)

Figure S2 **Mortality rates of larvae stabbed with fungi-contaminated minutin pins within 7 days with N = 630.**
(TIF)
