# Supplementary figures and images for: Effect of Fungal Colonization of Wheat Grains with Fusarium spp. on Food Choice, Weight Gain and Mortality of Meal Beetle Larvae (Tenebrio molitor)
Source: PLoS One. 2014 Jun 16;9(6):e100112. doi: 10.1371/journal.pone.0100112 (PMC4059719; doi:10.1371/journal.pone.0100112)

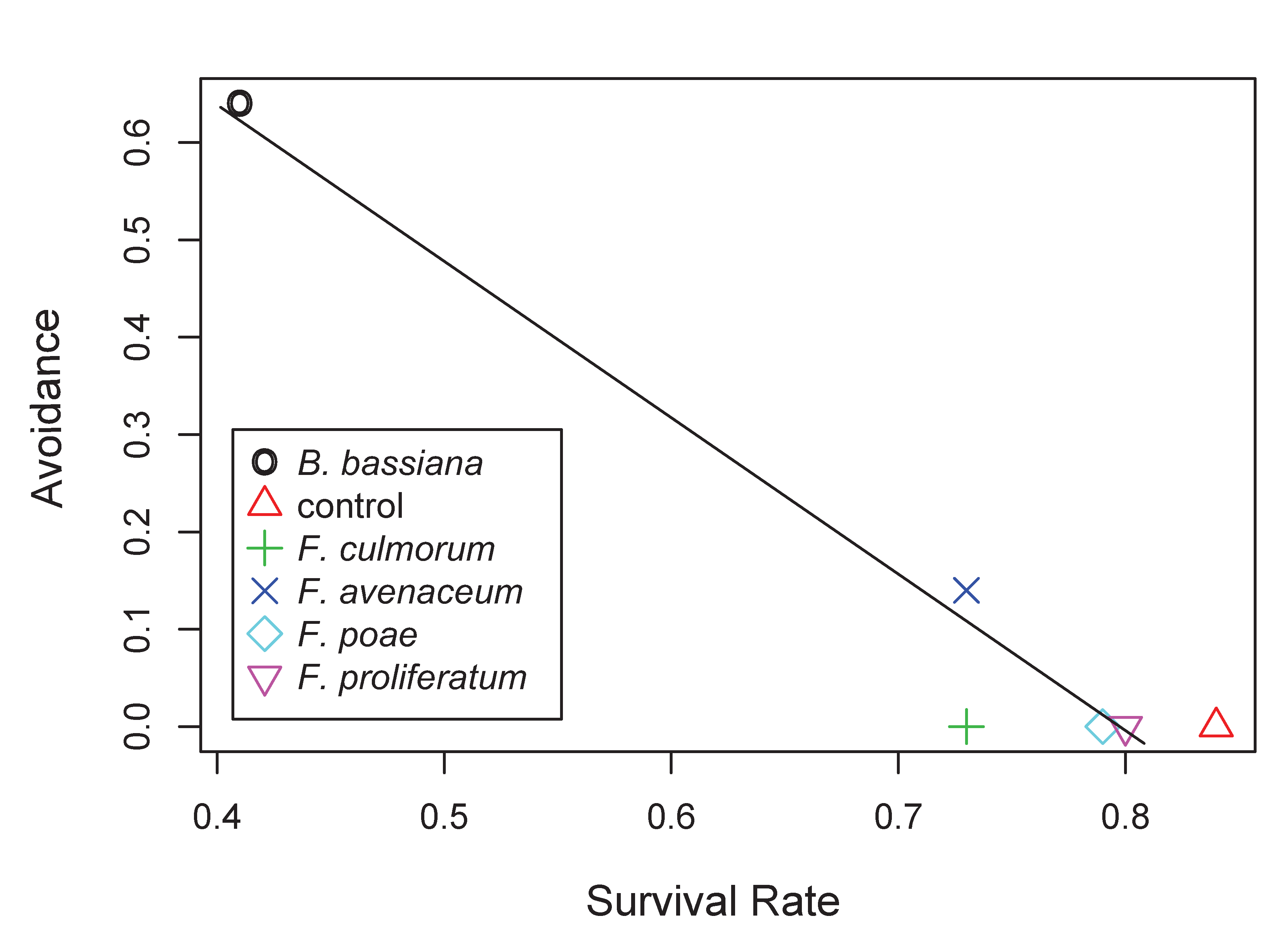

Supplement: Figure S1 — Correlation of avoidance levels of larvae towards fungi-colonized kernels to their respective survival rates. (TIF) [file pone.0100112.s001.tif]

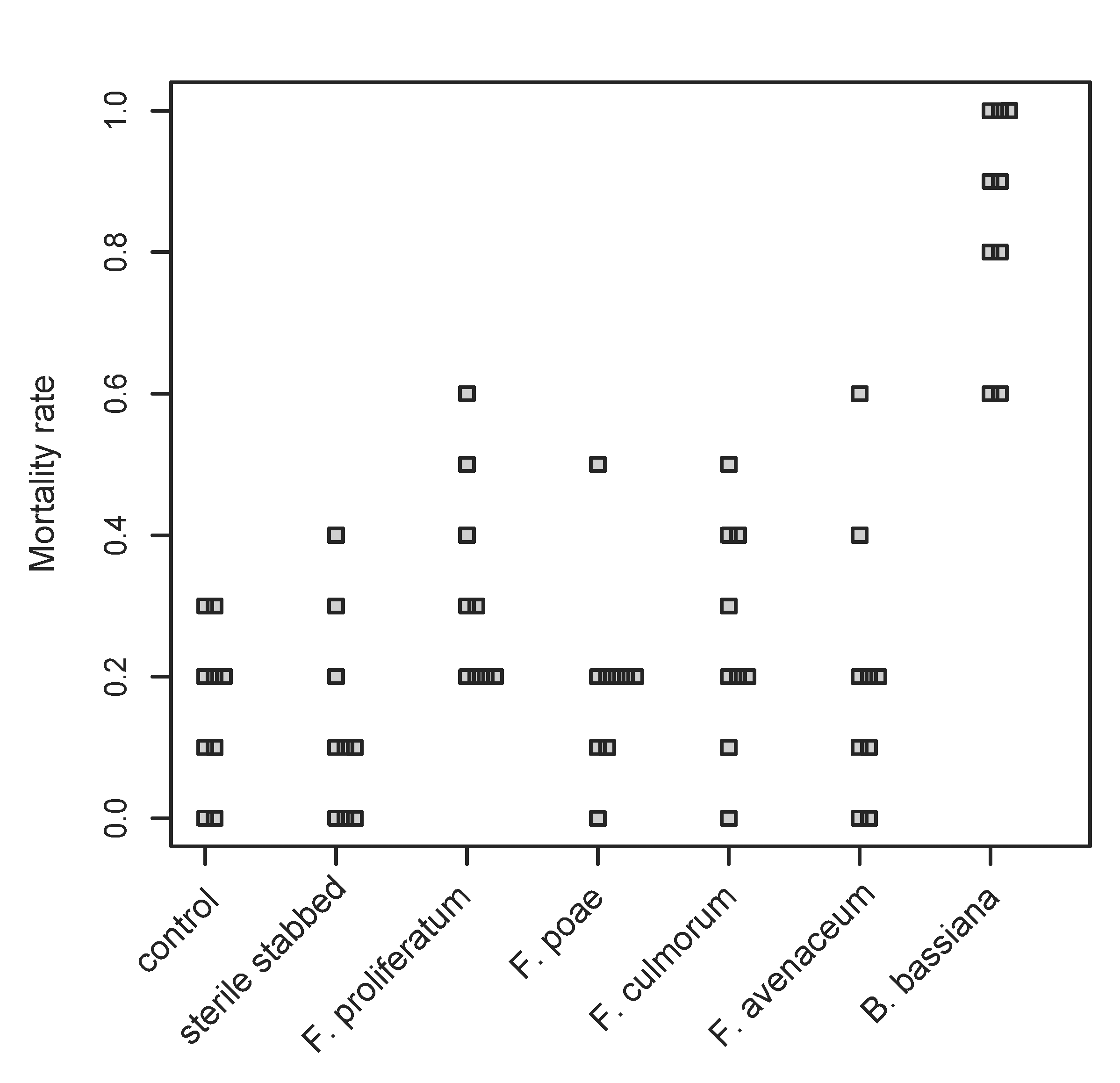

Supplement: Figure S2 — Mortality rates of larvae stabbed with fungi-contaminated minutin pins within 7 days with N = 630. (TIF) [file pone.0100112.s002.tif]
